# Identification of the first intragenic deletion of the *PITX2 *gene causing an Axenfeld-Rieger Syndrome: case report

**DOI:** 10.1186/1471-2350-7-82

**Published:** 2006-11-29

**Authors:** Guillaume de la Houssaye, Ivan Bieche, Olivier Roche, Véronique Vieira, Ingrid Laurendeau, Laurence Arbogast, Hatem Zeghidi, Philippe Rapp, Philippe Halimi, Michel Vidaud, Jean-Louis Dufier, Maurice Menasche, Marc Abitbol

**Affiliations:** 1 Centre de Recherche Thérapeutique en Ophtalmologie, EA n°2502, Faculté de Médecine René Descartes, site Necker, 156 rue de Vaugirard 75730 Paris cedex 15, Université Paris V, Paris, France; 2 Laboratoire de Génétique Moleculaire-INSERM U745, Faculté des Sciences Pharmaceutiques et Biologiques, Université Paris V, Paris, France; 3 CHU Necker Enfants Malades, Service d'ophtalmologie, 149, rue de Sèvres 75 Paris cedex 15, France; 4 Service de radiologie et d'imagerie médicale de Hôpital Européen Georges Pompidou, 20-40 Rue Leblanc, 75908 Paris Cedex 15, France

## Abstract

**Background:**

Axenfeld-Rieger syndrome (ARS) is characterized by bilateral congenital abnormalities of the anterior segment of the eye associated with abnormalities of the teeth, midface, and umbilicus. Most cases of ARS are caused by mutations in the genes encoding PITX2 or FOXC1. Here we describe a family affected by a severe form of ARS.

**Case presentation:**

Two members of this family (father and daughter) presented with typical ARS and developed severe glaucoma. The ocular phenotype was much more severe in the daughter than in the father. Magnetic resonance imaging (MRI) detected an aggressive form of meningioma in the father. There was no mutation in the *PITX2 *gene, determined by exon screening. We identified an intragenic deletion by quantitative genomic PCR analysis and characterized this deletion in detail.

**Conclusion:**

Our findings implicate the first intragenic deletion of the *PITX2 *gene in the pathogenesis of a severe form of ARS in an affected family. This study stresses the importance of a systematic search for intragenic deletions in families affected by ARS and in sporadic cases for which no mutations in the exons or introns of *PITX2 *have been found. The molecular genetics of some ARS pedigrees should be re-examined with enzymes that can amplify medium and large genomic fragments.

## Background

ARS is an autosomal dominant disorder characterized by bilateral congenital abnormalities of the anterior segment of the eye associated with abnormalities of the teeth, midface, and umbilicus. These defects may include microdontia, hypodontia, anodontia and maxillary hypoplasia. Classic ocular features of ARS include iridocorneal synechiae, iris hypoplasia, corectopia, polycoria, and/or prominent anteriorly displaced Schwalbe's line (posterior embryotoxon). Glaucoma develops in approximately 50% to 60% of patients with ARS. Axenfeld's and Rieger's anomalies were originally described as separate clinical entities. These and various abnormalities of the anterior chamber of the eye are now considered to fall into the same spectrum of developmental disorders, or even to be variations of ARS [1].

ARS has been linked to five chromosomal loci (4q25, 6p25, 11p13, 13q14, 16q24) [2-4] Disease-causing mutations have been identified in three transcription factor genes. Two of these genes – *PITX2 *and *FOXC1*, which map to chromosomes 4q25 and 6p25, respectively – are the most frequently affected. Only one case of ARS caused by deletion of the paired-box transcription factor, *PAX6*, which maps to chromosome 11p13 has been reported [5]. The causal genes at chromosome 13q14 and chromosome 16q24 loci have not been identified.

The *PITX2 *gene (OMIM: 601542) was identified by positional cloning of the chromosome 4q25 locus and has been implicated in ARS pathogenesis. The *PITX2 *gene has seven exons [6] and encodes a member of the bicoid/paired-like homeodomain family [7]. However, only 40% of patients diagnosed with classical Rieger syndrome have PITX2 mutations [4]. *PITX2 *haploinsufficiency may cause this syndrome [8,9]

PITX2 clearly plays an important role in embryonic and foetal development. PITX2 is also required for the normal development of neurons in the mouse midbrain [8,9] and is involved in pituitary gland development. Thus, brain MRI in patients with ARS is justified.

New PITX2 mutations causing ARS have been reported recently. More information can be found in the web database [10].

We investigated the molecular basis of a severe form of Axenfeld-Rieger Syndrome in one family for which no mutation of *PITX2 *or *FOXC1 *was found. Our findings from quantitative genomic PCR and additional experiments indicate that a 3,059 bp intragenic deletion in the *PITX2 *gene causes this unusual form of ARS.

## Case presentation

We analysed one family in which two patients were affected by an unusual form of ARS. The man (I-2) was 51 years old and presented with bilateral Axenfeld-Rieger syndrome with hypodontia, maxillary hypoplasia and redundant periumbilical skin. He had chronic glaucoma that had been monitored clinically and surgically for at least 10 years. During this period of follow-up, he had undergone bilateral trabeculectomy.

**Figure 1:**
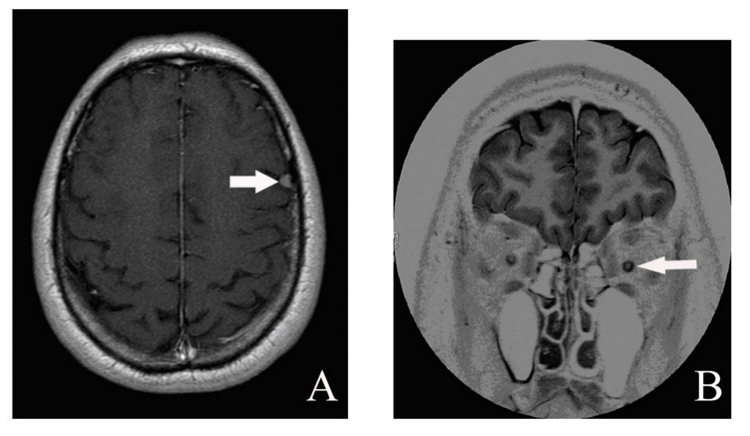
MRI scan for patient I-2. A) Left frontal meningioma measuring 4 mm in diameter. B) Atrophy of the two optic nerves in their intraorbital portions (before the intracanal portion) with dilatation of the sheaths.

An MRI scan carried out in June 2005 (Figure 1a) showed a left frontal meningioma that was 4 mm in diameter and atrophy of the two optic nerves in intraorbital sections. We observed marked dilatation of the myelin sheaths (Figure 1b). A second MRI scan in January 2006 showed that the meningioma had doubled in size over seven months.

This man also had severe thyroid problems with goiter consisting of bulky nodules that were up to 3 cm in diameter on both the right and left parts of the gland. He presented with hyperthyroidism that was not associated with pituitary adenoma or functional pituitary hormonal abnormalities.

**Figure 2:**
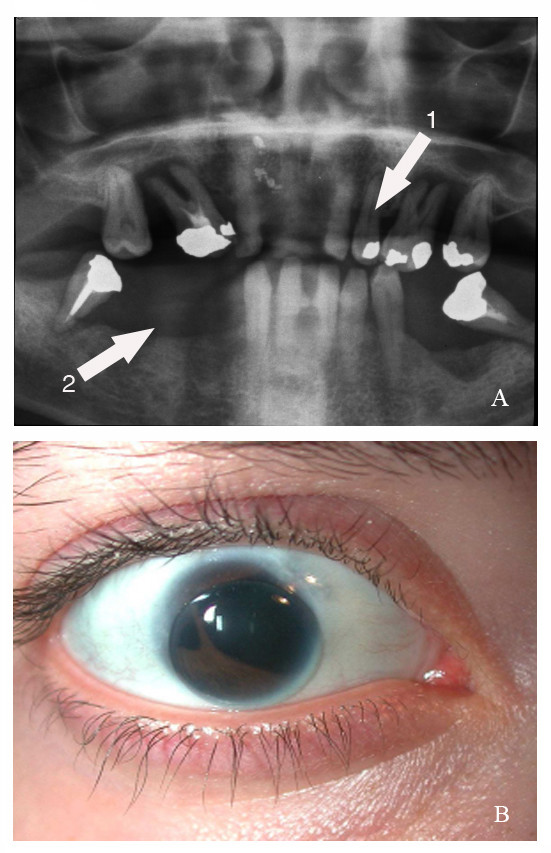
Phenotypic analysis of patient II-1. A) Panoramic dental X-ray showing hypodontia (arrow 1) and area of maxillary hypoplasia (arrow 2), B: Eye imaging showing polycoria.

This man's daughter (II-1) was 21 years old at the time of the study. She presented with the same characteristic features of ARS (Figure 2a and 2b), but had developed them earlier. She had severe glaucoma resulting in the early removal of the left eye and the fitting of a prosthesis. She presented with no abnormality of the optic nerves or retrochiasmatic visual pathways. Unlike her father, MRI scans showed she had no signs of meningioma or optic nerve abnormality.

**Figure 3:**
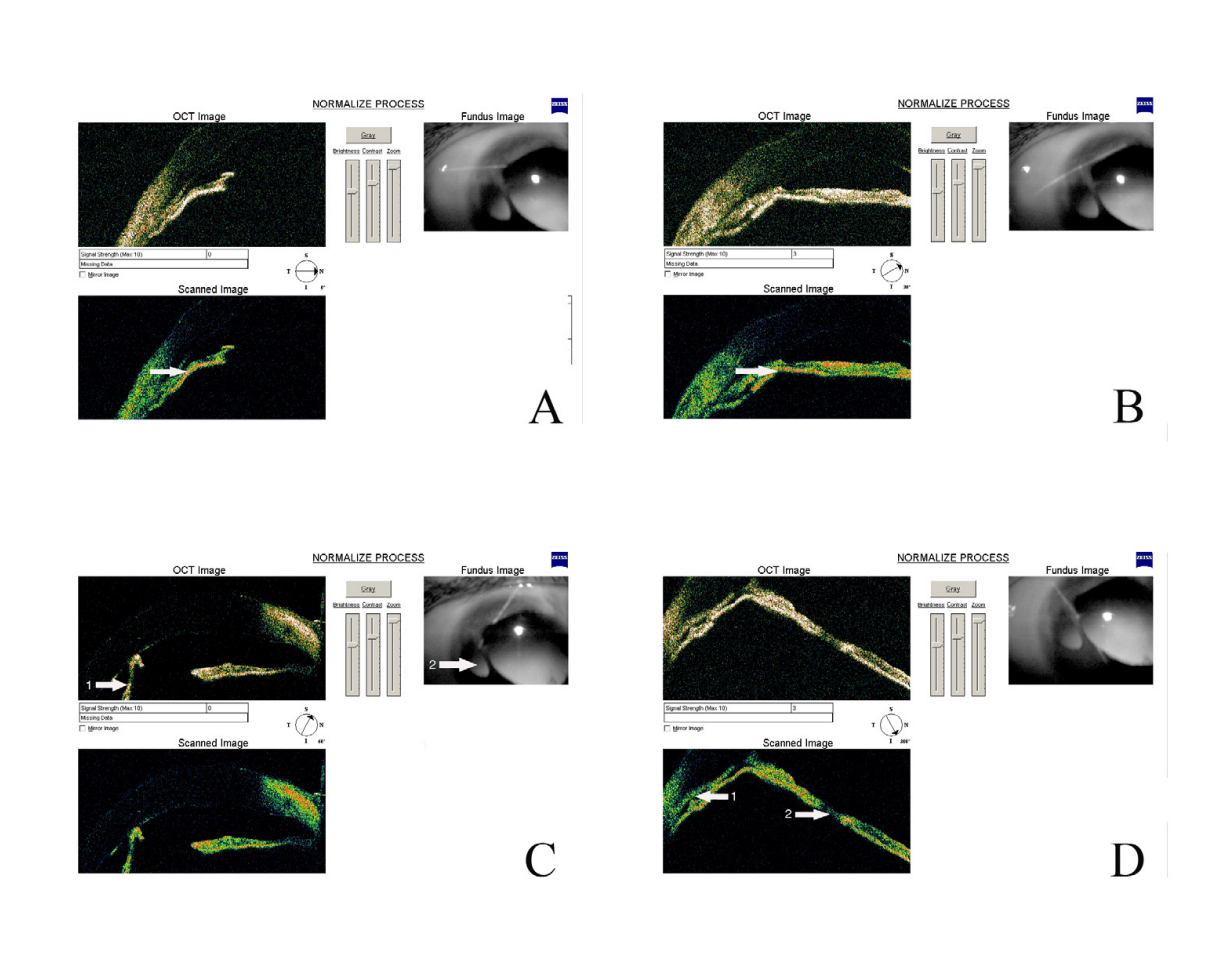
OCT examination of patient II-1. A) Partial iris apposition to the cornea, decreasing the iridocorneal angle of the right eye. Presence of abnormal goniostrands.(arrow) B) Abnormal goniosynechiae associated with goniostrands in a supratemporal position in the right eye (arrow). These structures significantly decrease the iridocorneal angle and clearly explain the very high intraocular pressure measured in this remaining eye. C) Polycoria with a heterogeneous iris and abnormal structure of the cornea. An iridocorneal synechia is visible between the corneal endothelium (arrow 1) and the inner part of the iris (arrow 2). It appears to be the remnant of iris hypoplasia and contributes to polycoria. D) Visualization of abnormal goniostrands (arrow 1) and of an abnormal remnant iris bridge effected by partial hypoplasia (arrow 2).

The father's visual acuity was reduced to 20/200 in both eyes, with only narrow parts of the visual field unaffected. The daughter had poor visual acuity in her remaining eye, limited to counting fingers. Slit lamp examination and photographic imaging showed polycoria associated with iridocorneal synechia and iris hypoplasia in both father and daughter (Figure 2b). We also observed polycoria by slit lamp examination and anterior optical coherent tomography (Figure 3) with an OCT3 Stratus machine that was defocused for anterior chamber examination. We saw goniostrands and parts of the iris that were very near the cornea in some sectors of the anterior chamber. This severely reduced the iridocorneal angle and accounted for the persistent high intraocular pressure (40 mmHg minimum value) measured with a Goldmann aplanation tonometer, despite previous trabeculectomy and ongoing maximal anti-glaucomatous medical treatment. Both patients were fully examined in the cardiology, pneumology and endocrinology departments of our hospital. Other than the thyroid abnormalities of I-2, which were not of pituitary origin and were treated, the patients displayed no abnormalities.

## Genetics analysis

### Characterization of a deletion in the *PITX2 *gene

**Figure 4:**
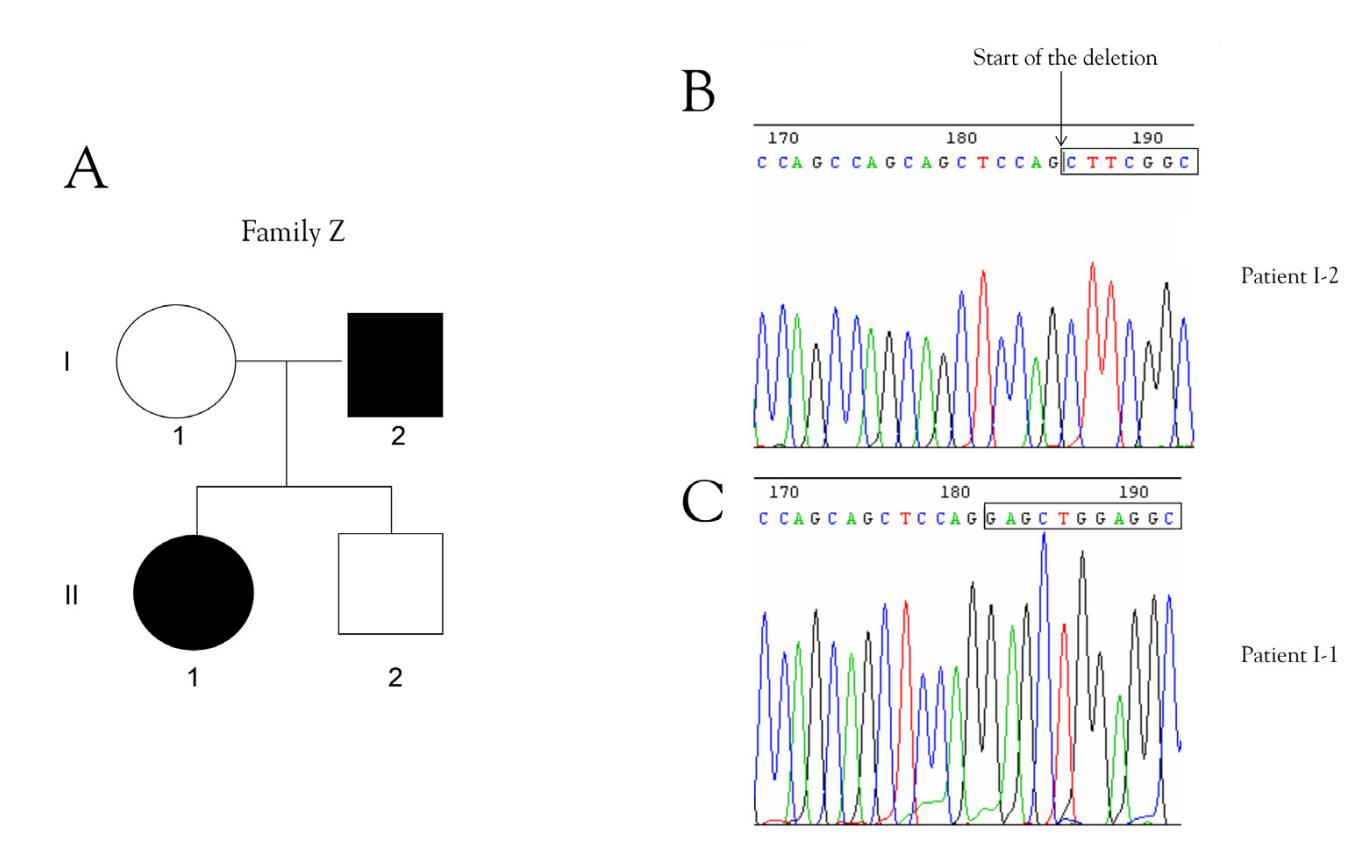
A) Genealogy of the family. B) Sequence of PITX2 in patient I-2. C) Normal sequence of PITX2 (patient I-1).

**Figure 5:**
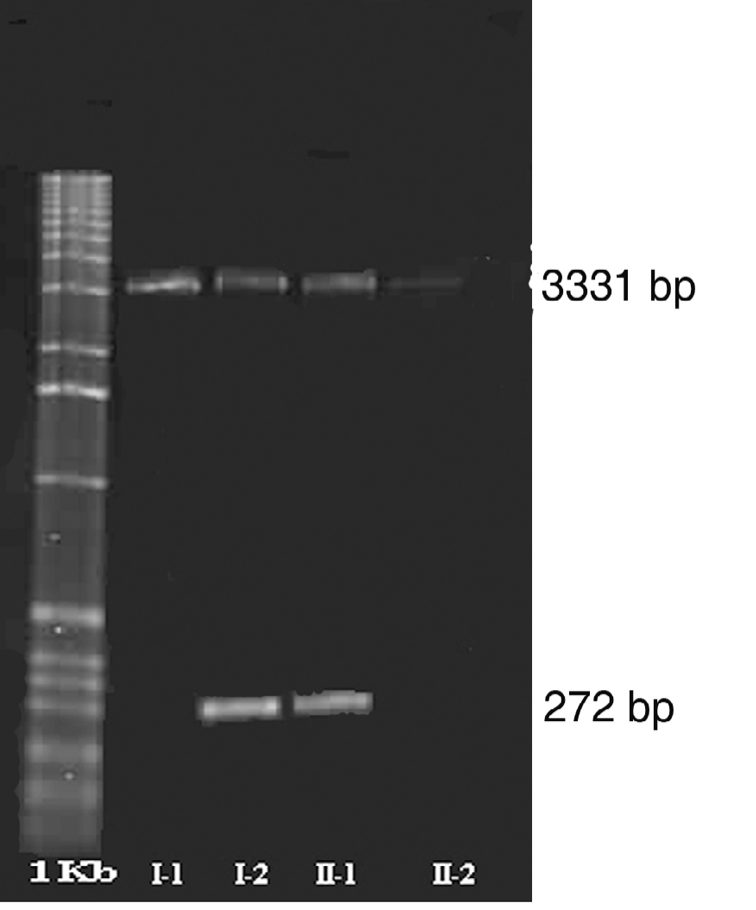
Agarose gel showing the deletion. The normal fragment is 3,331 bp in length. The abnormal fragment is 272 bp in length.

**Figure 6:**
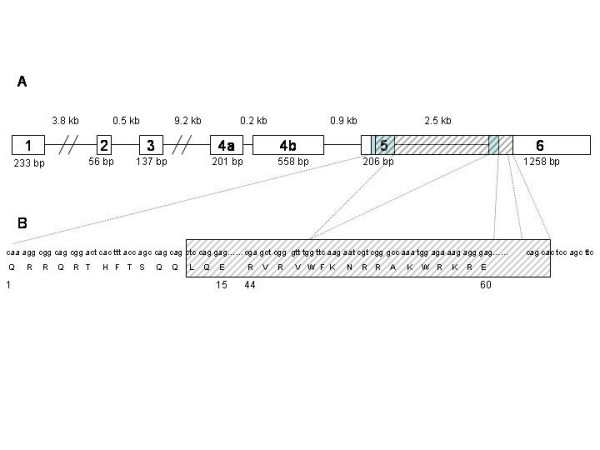
A) Schematic of *PITX2 *gene organisation. Boxes represent exons, lines represent introns. We have indicated the sizes of exons and introns. The blue box represents the homeodomain. The hatched box represents the deleted sequence. B) Nucleotide sequences and corresponding amino acids.

We looked in this family for *PITX2 *mutations (Figure 4a) that might account for the unusual phenotype. We screened the seven exons of the *PITX2 *gene by PCR with different primers [11] [see Additional file 1] and by sequencing, but found no mutation. We then searched for an intragenic deletion in the *PITX2 *gene by quantitative genomic PCR (Table 1) [12] with primer pairs designed for the amplification of both exon 5 and exon 6. Both these exons were present as a single copy. Each of the affected patients had only one copy of the *PITX2 *gene. We identified, in both patients, an intragenic deletion corresponding to part of exon 5, intron 6 and part of exon 6. We used two primers, one binding just before exon 5 (5'-CAGCTCTTCCACGGCTTCT-3') and the other binding just after exon 6 (5'-CTGTGGGTGCGGCTCACA-3'), to amplify a fragment of DNA large enough to determine accurately the start and end points for the intragenic deletion. We amplified only a 3,331 base-pair (bp) fragment from control genomic DNA from unaffected patients. However, we amplified the 3,331 bp fragment and an additional abnormal fragment of 272 bp from the genomic DNA samples from of the patients with ARS (I-2 and II-1). The short fragment corresponded to the genomic sequence of the abnormal partially deleted *PITX2 *gene (Figure 5). We characterized this deletion by sequencing the amplified fragments of genomic DNA from controls and affected patients (Figure 4b and 4c). The deletion began six nucleotides from the 5' end of exon 5 (5'-CTCCAG-3') and ended after the same repeated sequence in exon 6. Thus, 3,059 bp between the nucleotides 18,183 to 21,242 within the *PITX2 *gene (gi|13183092|gb|AF238048.1| [13183092]) were deleted. This deletion removed the end of exon 5, the following intron and the beginning of exon 6 (Figure 6). Consequently, the *PITX2 *genomic sequence spanning 144 of the 180 nucleotides of the homeobox sequence was lost. Should the gene be transcribed and the corresponding transcripts not degraded by nonsense-mediated mRNA decay, they might be translated. The resulting abnormal proteins would contain only the first twelve amino acids of the PITX2 homeodomain. The homeodomain is present in the PITX2 A, B and C isoforms and contains helix3, implicated in DNA binding. Thus, the truncated homeodomain, lacking helix3, would not be functional (Figure 6).

**Table 1:** Dosage of the *PITX2 *gene was determined by genomic quantitative PCR. We used *ALB *and *ERBB *as endogenous DNA control genes. *PITX2 *is expressed from only one copy in patients I-2 and II-1, in contrast to what was observed for other members of the family (I-1 and II-2) and unrelated cases (856, 23128, 31964).

	ALB		PITX2 U2/L2			
	
	n Ct	Δ	n Ct	Δ	PITX2/ALB	
I-1	21,1	118,6	21,9	8,8	0,7	1,1
I-2	20,8	144,3	22,6	5,2	0,4	0,5
II-1	20,5	180,4	22,7	4,9	0,3	0,4
II-2	21,5	90,5	22,3	6,6	0,7	1
856	21,6	86,2	22,3	6,5	0,8	1
23128	20,4	190,9	21,2	14,0	0,7	1
31964	20,5	179,1	2,4	12,5	0,7	1
	ERBB		PITX2 U2/L2			
	
	n Ct	Δ	n Ct	Δ	PITX2/ERBB	

I-1	21,1	122,8	21,9	8,8	0,7	1,0
I-2	20,8	150,1	22,6	5,2	0,3	0,5
II-1	20,8	145,0	22,7	4,9	0,3	0,5
II-2	21,4	95,7	22,3	6,6	0,7	1,0
856	21,5	92,0	22,3	6,5	0,7	1,0
23128	20,2	221,3	21,2	14,0	0,6	0,9
31964	20,8	151,9	2,4	12,5	0,8	1,1

### Consequences of the intragenic deletion on the *PITX2 *mRNA sequence

We investigated how the *PITX2 *intragenic deletion affected the *PITX2 *mRNA sequence. For this purpose, we sequenced *PITX2 *following RT-PCR from total RNA isolated from lymphoblastoid cell lines established from the two ARS-affected patients. The RT-PCR amplification was done with primers that were unambiguously outside the deletion area: P5, 5'-AGCGGACTCACTTTACCAGC-3', was in exon 5 and P6, 5'-CCCACGACCTTCTAGCATAA-3', was in exon 6. The amplified region is conserved in the three *PITX2 *mRNA isoforms both from the DNA samples of normal individuals and affected patients. The amplification yielded one fragment of the expected size. No abnormal fragments were detected (data not shown). Thus, our findings support the notion that simple *PITX2 *haploinsufficiency causes the pathogenicity of the intragenic *PITX2 *microdeletion in the eye and possibly in other target tissues.

## Discussion

We have analysed a family affected by an unusual form of ARS. The difference in the phenotypes between I-2 and II-1 is typical for this syndrome. A severe, acute increase in intraocular pressure in II-1 had an effect similar to axotomy, with no optic nerve damage visible by MRI. However, a chronic increase in intraocular pressure that progressed over a long period of time in I-2 triggered the progressive disappearance of axons in the optic nerve, with enlargement of the myelin sheath (Figure 1b) which may have provided a degree of neuroprotection.

The atrophy of the optic nerve that was observed by MRI in the father has not been reported previously in patients with ARS. However, a recent study provides a possible explanation for this observation. Mice in which the *pitx2 *gene has been specifically knocked out in the neural crest have optic nerve atrophy [13]. This suggests that PITX2 is involved in the formation of the optic nerve and/or its susceptibility to high intraocular pressure. Together with the findings of Ittner and al. and Berry and al [14,15], these results strongly support the hypothesis that PITX2 regulates levels of extrinsic factor(s) required for optic nerve development.

No other case of meningioma in association with ARS has been reported. The occurrence of both meningioma and ARS in I-2 may reflect an unlikely coincidence with these conditions arising through different mechanisms. Patient II-1, carrying the same mutation, had no signs of meningioma when she was examined. We need to determine whether this clinical observation results from deletion of the *PITX2 *gene or whether it is an independent event. Various meningiomas have been associated with the translocation of part of chromosome 4 to chromosome 22 [16], but no direct relationship with the *PITX2 *gene was found.

Meningiomas are usually benign intracranial and intraspinal tumors. However, invasive meningiomas can penetrate the brain parenchyma and disturb vital structures. Meningioma cells are derived from the craniofacial neural crest, which migrates around the anterior neural tube and colonizes the head mesenchyma [17,18]. Therefore, meningiomas have been suspected for a long time to express genetic programmes similar to the foetal meninges. The mouse ortholog of *FOXC1*, called *Mf1*, is expressed strongly in the meninges [19,20]. Recently, PITX2A was reported to function as a negative regulator of FOXC1 transactivation activity with its homeodomain [15]. The *FOXC1 *gene expression pattern is modified in several human cancer cell lines [21-23]. A significant fraction of primary cancers displays somatic mutations in this gene encoding a member of the Forkhead transcription factor family. Thus, FOXC1 may function as a tumour suppressor gene, through TGF-β1-mediated signals [24]. The *FOXC1 *transcription factor gene is critical for the formation of tissues derived from neural crest and mesenchymal mesoderm cell lineages [20,25]. It was recently reported that FOXC1 was associated with tumours originating from the mesenchyme including synovial sarcomas [22]. The *FOXC1 *gene and some of its target genes involved in the TGFβ-1 pathway appear to be upregulated, as shown by microarray experiments and RT-qPCR [22]. PITX2 serves as a competence factor required for the temporally-ordered and growth factor-dependent recruitment of a series of specific coactivator complexes necessary for cyclin D2 and cyclin D1 [26] gene induction. *PITX2 *gene expression has been associated with cancerogenesis. It was recently suggested that increases in *PITX2 *gene expression leads to the nuclear accumulation of β-catenin in the nuclei of pituitary cells, leading to malignant adenoma [27,28]. *PITX2 *is also a target gene for the product of the mixed lineage leukemia (MLL) gene [29]. The protein encoded by the *MLL *gene is directly involved in acute leukaemia associated with abnormalities in chromosome 11q23 in humans [30].

Now it appears that the amounts of the *PITX2 *and *FOXC1 *genes expressed are crucial for triggering developmental or oncogenic abnormalities. Many lines of evidence have identified *PITX2 *and *FOXC1 *as genes possibly involved, primarily or secondarily, in the multistep processes of cancerogenesis. Further studies should clarify the roles for FOXC1 and PITX2 in human developmental processes and oncogenesis [31]. Unexpected interactions between PITX2 and FOXC1 proteins have been recently discovered [15]. Currently, the molecular basis for ARS clinical manifestations is thought to involve both increased and decreased PITX2 and FOXC1 activity. Protein interactions may explain the strict dosage sensitivity of *PITX2 *and *FOXC1*: PITX2-PITX2 and PITX2-FOXC1 complexes may form in a concentration and/or cofactor-dependent fashion. Changes in the expression of either gene may alter the relative abundance of both types of complex. If PITX2-PITX2 and PITX2-FOXC1 complexes are mutually exclusive, they may interact with distinct cofactors and/or activate different groups of target genes. Alternatively, PITX2-PITX2 and PITX2-FOXC1 complexes may compete for a common pool of transcriptional cofactors and/or binding sites. The deletion identified in the family presented here affected the C-terminal domain of PITX2 and is likely to affect FOXC1 activity. Regulation through FOXC1-PITX2 interactions is complex. Thus, *FOXC1/PITX2 *mutations or deletions may lead to the transcriptional dysregulation of a subset of target genes and not only the loss of target gene expression by either *PITX2 *or *FOXC1 *alone. Thus, depending on the cellular context, genetic alterations of *FOXC1 *or *PITX2 *could result in the loss of transcriptional activation or the loss of transcriptional repression of target gene activity [15].

We did not detect aberrant forms of *PITX2 *in lymphoblastoid cell lines or in white blood cells. However, this does not mean that the mutated *PITX2 *allele is not expressed *in vivo*. The homeodomain of the abnormal PITX2 proteins presumably produced in the affected patients might include the first twelve amino acids of this domain. Normally, the PITX2 protein interacts with the FOXC1 protein through its paired homeodomain. However, the PITX2-FOXC1 interaction may not occur or could be very loose in these ARS patients. The aberrant PITX2 proteins resulting from the possible translation of the shortened *PITX2 *mRNA isoforms might form complexes with normal FOXC1 proteins and would not be able to impair FOXC1 activity. This would explain the severe phenotype observed in both affected patients and the occurrence of a meningioma in patient I-2.

FOXC1 seems to participate in several transcription factor complexes including PBX1 [35]. Thus, it appears that the pathophysiology of ARS and the diversity of clinical manifestations observed in ARS-affected patients is caused by complex abnormal transcriptional mechanisms and abnormal (post) translational interactions between several transcription factors and between the DNA/transcription factor complexes. Whatever the cause of the meningioma in patient I-2 and potentially in patient  II-1, all patients with ARS should be monitored regularly by brain and spine MRIs.

The most common cause of ARS is a mutation in the *PITX2 *gene or in the *FOXC1 *gene. However, exon-by-exon screening for mutations in genomic DNA may fail to detect a mutant allele. This is illustrated by the case reported here. There were no mutations detected in the seven exons and adjacent introns of the *PITX2 *gene. The most probable explanations for the apparent absence of *PITX2 *gene alteration are that a large DNA rearrangement was masked by the presence of the wild-type *PITX2 *normal allele or that there was an undetected mutation in an intron. The absence of clinical abnormalities in the previous generation (data not shown) suggests that this may be a *de novo *genomic alteration. Unfortunately, the grandparents of patient II-1 were not available for participating in this study.

Following the detection of an intragenic *PITX2 *deletion by quantitative genomic PCR, we developed a PCR protocol for amplifying exon 5, intron 6 and exon 6 to look for DNA rearrangement. Fine mapping of the deletion breakpoint by direct sequencing of the PCR products showed that the deletion had removed a 3,059 bp region extending from the end of exon 5 to the start of exon 6. The junctions at the beginning and end of the deletion contained a short direct six bp repeat (CTCCAG). This is not the first time that quantitative genomic PCR has been used to detect *PITX2 *deletions. However, only microdeletions and gross deletions without refined molecular characterization have been reported [32]. The deletions reported previously were not molecularly characterized and might correspond to closely related syndromes [32]. Some involved the loss of several genes and resembled the interstitial microdeletions detectable by FISH. This is the first report of an entirely intragenic *PITX2 *deletion, accurately characterized, that does not lead to complete *PITX2 *gene deletion. There are various possible explanations for this intragenic deletion. All the proposed mechanisms for deletions or other rearrangements in human genetic disorders – including genomic disorders, a subset of genetic disorders associated with large deletions and/or duplications – are associated with the presence of repetitive sequences. One of these mechanisms is the slipped mispairing hypothesis, first proposed by Streisinger [33] and initially observed in yeast. The CTCCAG of exon 5 could bind to the corresponding GAGGTC sequence in exon 6 on the other strand. This would result in the formation of a single-stranded loop that could be excised by a DNA repair enzyme, for example RAD [34]. Slipped mispairing is probably the mechanism for the deletion reported in this study (data not shown). However, we cannot rule out the possibility that non-homologous end joining (NHEJ) led to this deletion. The intragenic deletion in this family resembles the haploinsufficency effects of most *PITX2 *mutations causing ARS [1].

## Conclusion

Mutational status has been reported for many patients with ARS. However, the overall detection rate for mutations is low. This is the first time that an intragenic deletion of the *PITX2 *gene has been identified, with a PCR protocol designed to amplify larger genomic fragments than those usually amplified for direct sequencing. The detection and the characterization of such a deletion were made possible by initial data provided by quantitative genomic PCR. Genomic fragments of one to several kilobases can be amplified with common Taq polymerase enzymes with intron-spanning primers in classical PCR reactions, facilitating the detection of medium sized intragenic deletions. The advent of new Taq polymerases (TaKaRa La Taq enzyme Cambrex) for long-range PCR and quantitative genomic PCR provide new tools for detecting large intragenic deletions throughout an entire gene. These tools, normal genomic PCR with intron spanning primers, and restriction enzymes will facilitate the refinement of *PITX2 *mutation analysis and ARS molecular diagnosis. They should also increase the sensitivity of genomic mutational screening. The screening of genes upstream or downstream from *PITX2 *or *FOXC1 *in ARS-affected families or in sporadic cases of ARS remains to be explored.

## Abbreviations

ARS: Axenfeld-Rieger syndrome

PITX2: paired-like homeodomain transcription factor 2

PCR: polymerase chain reaction

MRI: magnetic resonance imaging

bp: base pair

DNA: deoxyribonucleic acid

RNA: ribonucleic acid

FOXC1: forkhead box C1

MLL: mixed lineage leukaemia

## Competing interests

The author(s) declare that they have no competing interests.

## Authors' contributions

GH sequenced the patients' DNA, characterized the deletion and drafted the manuscript; VV and LA helped with the experiments. IB, IL and MV did the quantitative genomic PCR. HZ, PR, OR, JLD and MA provided ophthalmic diagnoses, supplied patient DNA and participated in the editing of the manuscript. PH did the MRI analysis. MM and MA designed and supervised the study. MA also helped draft the manuscript. All authors read and approved the final manuscript.

## Pre-publication history

The pre-publication history for this paper can be accessed here:



## Supplementary Material

Additional File 1 List of primers. List of primers used and the expected PCR product size Click here for file
