# Supplementary material for: Identification of the first intragenic deletion of the PITX2 gene causing an Axenfeld-Rieger Syndrome: case report
Source: BMC Med Genet. 2006 Nov 29;7:82. doi: 10.1186/1471-2350-7-82 (PMC1684248; doi:10.1186/1471-2350-7-82)
Supplement: Additional File 1 — List of primers. List of primers used and the expected PCR product size [file 1471-2350-7-82-S1.doc]

| exons | primers forward 5’-3’ | primers reverse 5’-3’ | *amplicon size (bp)* |
| --- | --- | --- | --- |
| exon 1 | TTGGCTCCTAAGTGCCCC | CCAGACTCGCATTATCTCAC | *596* |
| exon 2 | TAGTCTCATCTGAGCCCTGC | CACTGGCGATTTGGTTCTGA | *282* |
| exon 3 | ACGCCTCTCTCCGCACGT | TTCTTGCGCTTTCGCCCGA | *258* |
| exon 4 (a+b) | CTTGACACTTCTCTGTCAGG | AAGCGGGAATGTCTGCAGG | *667* |
| exon 5 | CAGCTCTTCCACGGCTTCT | TTCTCTCCTGGTCTACTTGG | *374* |
| exon 6 up | GTAATCTGCACTGTGGCATC | CTGTGGGTGCGGCTCACA | *627* |
| exon 6 down | CTGAGACTGAAAGCAAAGCA | CTCCCATGAAATAAAACACATTT | *787* |
| exon 5 + 6 | CAGCTCTTCCACGGCTTCT | CTGTGGGTGCGGCTCACA | *normal size :3331*  *delete sequence :272* |
| arn | AGCGGACTCACTTTACCAGC | CCCACGACCTTCTAGCATAA | *762* |
| U2/L2 | GCCAGCCTGAGACTGAAAGCA | GCATACTGGCAAGCACTCAGGTT | *71* |
| ALB | GCTGTCATCTCTTGTGGGCTGT | ACTCATGGGAGCTGCTGGTTC | *139* |
| *ERBB2* | *AGCCGCGAGCACCCAAGT* | *TTGGTGGGCAGGTAGGTGAGTT* | *147* |
